# A computational method to predict topologically associating domain boundaries combining histone Marks and sequence information

**DOI:** 10.1186/s12864-019-6303-z

**Published:** 2019-12-27

**Authors:** Wei Gan, Juan Luo, Yi Zhou Li, Jia Li Guo, Min Zhu, Meng Long Li

**Affiliations:** 1 College of Computer Science, Sichuan University, Chengdu, 610064 People’s Republic of China; 2 College of Chemistry, Sichuan University, Chengdu, 610064 People’s Republic of China; 3 College of Cybersecurity, Sichuan University, Chengdu, 610064 People’s Republic of China

**Keywords:** Histone modification, Topologically associated domains, Deep learning, Sequence information

## Abstract

**Background:**

The three-dimensional (3D) structure of chromatins plays significant roles during cell differentiation and development. Hi-C and other 3C-based technologies allow us to look deep into the chromatin architectures. Many studies have suggested that topologically associating domains (TAD), as the structure and functional unit, are conserved across different organs. However, our understanding about the underlying mechanism of the TAD boundary formation is still limited.

**Results:**

We developed a computational method, TAD–Lactuca, to infer this structure by taking the contextual information of the epigenetic modification signals and the primary DNA sequence information on the genome. TAD–Lactuca is found stable in the case of multi-resolutions and different datasets. It could achieve high accuracy and even outperforms the state-of-art methods when the sequence patterns were incorporated. Moreover, several transcript factor binding motifs, besides the well-known CCCTC-binding factor (CTCF) motif, were found significantly enriched on the boundaries.

**Conclusions:**

We provided a low cost, effective method to predict TAD boundaries. Above results suggested the incorporation of sequence features could significantly improve the performance. The sequence motif enrichment analysis indicates several gene regulation motifs around the boundaries, which is consistent with TADs may serve as the functional units of gene regulation and implies the sequence patterns would be important in chromatin folding.

## Introduction

The spatial organization of the chromatin plays a key role in cellular processes [1], such as gene regulation, DNA replication and VDJ (variable, diversity and joining genes) recombination [2–4]. The development of techniques for the chromatin conformation capture, such as Hi–C, has been a significant breakthrough in understanding the genome-wide chromatin structure. The most important discovery of 3D (three-dimensional) genome studies are possibly the hierarchical structures: compartments A or B [5], topologically associated domains (TADs) [6, 7] and chromatin loops [8, 9], which shape the genome and contribute to the functioning of the genome [10]. The chromatin loops have been found to vary widely [8, 11]. As for the compartments, they are cell-type specific, but could not comprehensively describe differences between cell types across the genome [5]. In contrast, TADs, generally composed of many loops, being invariant and conservative during differentiation across cell types and tissues [7, 12], even between different species [2, 7, 11].

TADs are ubiquitous across the genome sequence near the diagonal in contact maps, but not seen at large genomic distances greater than a few mega bases. There are two basic features for the structure organization as a result of colocalization of the TADs [13]: self-association and insulation. The sequences within a TAD would preferentially contact with each other [6, 7, 14]. The enhancers and promoters of genes are found within a TAD and genes located in the same TAD can be activated simultaneously. Corresponding to the two basic features of organization, co-regulation and blocking of chromatins are two functional features of TADs. It was found to align with coordinately-regulated gene clusters in the mouse X-inactivation center [15]. This suggests that TADs may serve as the functional units of gene regulation [6]. It is not surprising that several studies suggest the disruption of this structure may cause diseases [15, 16]. It is therefore desirable to identify the TADs loci, as well as unravel their formation mechanisms, although this remains a remarkable challenge.

For this task, DomainCaller (DI) was first created to determine the location of TAD boundaries [7]. Other similar methods were also proposed, such as HiCseg [17], Armatus [18], CITD [19] and TADtree [20]. They are all fully dependent on the interaction frequency matrix derived from the Hi–C [7]. The interaction frequency matrix is an adjacency matrix for measuring the spatial distance between fragments on the genome. Due to the high cost and low resolution of the Hi–C experiments [20, 21]. An alternative strategy was proposed to infer TADs by using the histone mark patterns around TAD boundary and non-boundary [13], including the HubPredictor [21], PGSA [22] and nTDP [23]. HubPredictor only used eight histone and CTCF mark signals and did not take the up/down environment into consider. Although PGSA considered more than 10 gene elements, feature type is relatively single. Therefore, their performance was still unsatisfactory. The resolution of data is another aspect to investigate TAD boundaries [24], the mentioned methods did not show the impact of data resolution on their models.

Chromatin associated factors, such as CTCF and cohesins, recruit enhancers to their target genes. They are regarded as vital elements for shaping the genome. Some DNA sequences have a preference [25]. We therefore incorporate sequence information with the histone mark patterns and propose TAD–Lactuca to predict the TAD boundaries. We used the contextual information of the loci as inputs to explore patterns of CTCF and eight histone mark signals as well as k-mer’s frequency [26] between the boundaries and non-boundaries. Moreover, various resolutions were also investigated. Both random forest and deep learning algorithm were applied in our method. Our method is stable in various resolutions and different datasets. It could achieve high accuracy and even outperforms the state-of-art method when the sequence patterns incorporated. Moreover, several transcript factor binding motifs, beside the well-known CCCTC-binding factor (CTCF) motif, were found significantly enriched on the boundaries. A python 3.* implementation of the TAD–Lactuca and instructions for use are available at https://github.com/LoopGan/TAD-Lactuca.

## Results

### Signal patterns around the TAD boundaries

We firstly investigated the CTCF and histone mark signal patterns around TAD boundaries, including H3K4me1, H3K4me2, H3K4me3, H3K9ac, H3K9me3, H3K27ac, H3K27me3 and H3K36me3. We calculated the signal intensities under various resolutions for each feature. Two terms were employed to describe a locus and its chromatin context: *bin* _ *size* and *bin* _ *number*. Then, *Len*(*region*) can be calculated as the Eq. (1):

1 $$ Len(region)= bin\_ size\ast \left( bin\_ number\ast 2+1\right) $$

**Fig. 1:**
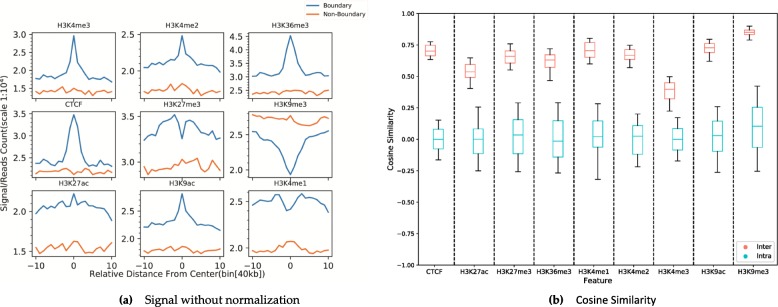
Histone mark signatures of TAD boundaries: **a** Non-transformed signal patterns, which were higher in the boundary compared to the non-boundary area. ‘0’ is the boundary bin, − 10 and 10 represent the number of bin distance with the center bin. ‘-‘ stands for upstream and ‘+’(ignored) stands for downstream bin. Y axis means each bin’s histone or CTCF modification intensity. **b** The cosine similarity of different signals between the TAD boundary and non-boundary areas. The inter-type is calculated from inter-category samples and the intra-type is calculated from intra-category samples, respectively

The *bin* _ *size* = 40*kb* and *bin* _ *number* = 10 resulted in a region of 840*kb*. We use this as an example to compare the enrichment difference of CTCF and eight different histone mark signals around the TAD boundaries and non-boundaries (Fig. 1).

The depletion of H3K9me3 around the TAD boundary was not present in non-boundary areas. It suggests that for a region with a similar Hi–C contact frequency, a stronger H3K9me3 mark signal intensity means that it is less likely to be a TAD boundary. This is because the H3K9me3 signal is usually associated with silenced genes [27]. At the boundary, the transcription may not be strong, most of the loci may be silenced genes. We also noticed that the signals of H3K4me1 and H3K27me3 are different from other signals. The H3K4me1 mark is positively correlated with the levels [27], with the TAD boundary having lower transcriptional levels compared with other regions in a TAD. The H3K27me3 mark signals were enriched at silent promoter regions, while they were reduced at active promoter regions and genic regions [27]. Therefore, these signals might be enriched around the TAD boundary instead of the center region of the TAD boundary.

**Fig. 2:**
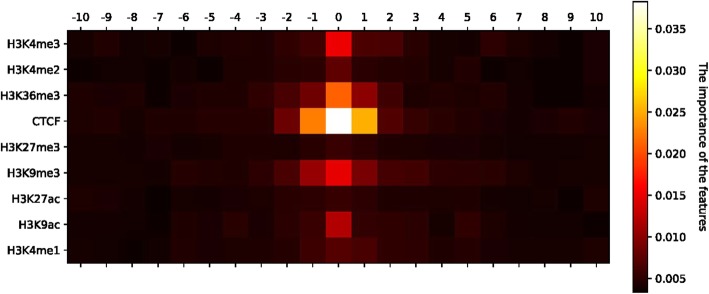
Heatmap of each bin’s importance, which was calculated by the function *feature_importance_* of sklearn [29]. The lighter the color of bins, the higher the importance

To evaluate the differences in CTCF and eight histone mark signals between the TAD boundaries and non-boundaries, we calculated the cosine similarity [28] of the two categories. The cosine similarity is calculated as follows:

2 $$ Sim\left(\overrightarrow{TAD},\overrightarrow{NonTAD}\right)=\frac{\sum_{i=1}^N\left({\overrightarrow{TAD}}_i\times {\overrightarrow{NonTAD}}_i\right)}{\sqrt{\sum_{i=1}^N{\overrightarrow{TAD}}_i^2}\times \sqrt{\sum_{i=1}^N{\overrightarrow{NonTAD}}_i^2}} $$

where the $\overrightarrow{TAD},\overrightarrow{NonTAD}$ denote the histone mark signal vector for a TAD boundary and a non-boundary, respectively. *N* represents the dimension of each vector. When we calculated the cosine similarity, each sample was processed with z-score standardization by factor type. In Fig. 2, we found that the mark signals within the same category always have significantly higher similar scores (Wilcoxon rank sum test, *p*-value < 0.05) than from different categories. In particular, for the CTCF mark signal, we observed that the cosine similarities are concentrated in (− 0.1, 0.1). The value of the intra cosine similarity was greater than the inter cosine similarity. This further suggest the mark patterns could be discriminative between TAD boundaries and non-boundaries.

### Sequence pattern analysis around the boundaries

Sequence patterns were also analyzed by performing motifs enrichment detection at TAD boundaries. Several chromatin structure and gene regulatory associated motifs were detected, such as CTCF, CAMTA, ERF3 and HINFP. Among them, the CCCTC-binding factor (CTCF) is a well-known chromatin protein, which organizes the higher-order chromatin structure and plays a key role in intrachromosomal/interchromosomal interactions [30]. CAMTA functions as a transcriptional activator and coactivator. It could control the cell growth and proliferation, and may function as tumor suppressors and in episodic memory performance [31]. The eukaryotic releasing factor ERF3 is a multifunctional protein that plays pivotal roles in translation termination as well as the initiation of mRNA decay [32]. ERF3 also participates in cell cycle regulation and cytoskeletal organization apart from its function in translation [33]. ERF3 also functions in the regulation of apoptosis [32]. The histone gene transcription factor HINFP is an essential developmental regulator of the earliest stages of embryogenesis, controlling H4 gene expression in early preimplantation embryos in order to support normal embryonic development [34].

### TAD boundary prediction

Besides the sequence information, nine protein factors were combined into TAD–Lactuca. To evaluate the prediction performance of different factors, we measured the importance of different bins and the performance of different features at *bin* _ *size* = 40*kb* and *bin* _ *number* = 10. Figure 2 shows the importance of different bins. TAD–Lactuca used the Gini Importance to evaluate the importance of each bin. Figure 2 shows that the bin located in the center of the region was the important feature. After we separated the nine types of features, we observed that the CTCF is the most important compared to other histones (Supplementary Materials). The central bins of the region indicate that the CTCF plays a dominant role and is the most predictive protein for distinguishing between the TAD boundary and non-boundary. This is consistent with the findings of previous studies [35–37]. Acting as enhancer blocking, CTCF can act as a chromatin barrier by preventing the spread of heterochromatin structures [38]. The CTCF binding sequence elements can block the interaction between enhancers and promoters. These two are consistent with the result of our model.

Random Forest was applied to the CCCTC-binding factor (CTCF), eight types of histone marks and also the sequence information (details in the section of ***Materials and Methods***), respectively. Then, the TAD–Lactuca was constructed by incorporating all these features. CTCF could well discriminate the TAD boundaries from non-boundaries with an averaged AUC value of 0.754 at five-fold cross-validation. When on the histone marks, the AUC was 0.773. The combination of these two types of features obtained an AUC value of 0.817. The sequence features, 3-mer, got the AUC of 0.636. All features incorporation could improve the AUC to 0.867. The MLP was similarly applied. Its performance was listed in Table 1.

**Table 1:** Prediction accuracy using various features and some combinations, with the AUC scores of different models shown in the table (TAD–Lactuca_RF represent Random Forests Model and TAD–Lactuca_MLP represents Multi-Layer Perceptron, the details of them are introduced at section 3.2.3.)

Methods	Features				
	ALL	CTCF+Histones	CTCF	Histones	3-Mer
HubPredictor	–	0. 774	0.703	–	–
TAD–Lactuca_RF	0.867	0. 817	0. 754	0. 773	0.636
TAD–Lactuca_MLP	0.812	0. 810	0. 752	0. 756	0.592

**Fig. 3:**
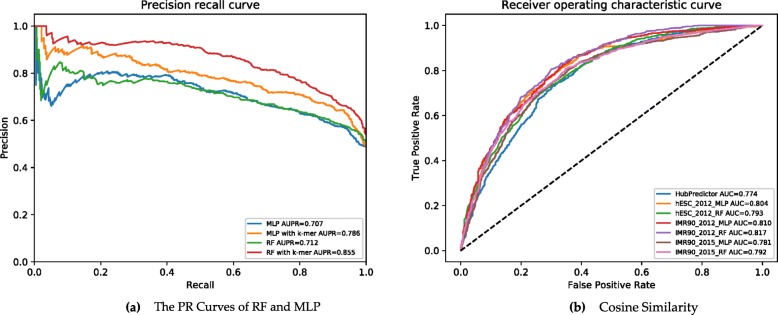
The result of TAD-Lactuca. **a** The Precision recall curves of RF and MLP. RF and MLP represent model only with histone and CTCF feature, RF with k-mer and MLP with k-mer represent model with sequence information respectively. **b** The ROC Curves among different datasets. 2012 in the legend means the dataset is from Dixon [7] and 2015 means the dataset is from Filippova [18]. For example, hESC_2012_MLP means the result of our MLP model on the dataset of Dixon [7]. AUC scores are shown in the legend

To illustrate the effectiveness of our method (TAD–Lactuca), a comparison was performed with HubPredictor [21] and PGSA [22]. Compared with HubPredictor [21], both TAD–Lactuca_RF(short as RF) and TAD–Lactuca_MLP(short as MLP) could achieve higher AUC than the HubPredictor (Table 1). Particularly when the sequence information incorporated, over ~ 0.1 higher AUC value was improved by RF. We also calculated AUPR (The area under the precision-recall curve) values, a common classifier evaluation index [39, 40]. Figure 3a shows the AURP values of different features combination of RF and MLP model. RF with k-mer gets the highest performance among them, which AURP was 0.855. Without the k-mers’ frequency, the performance will degrade. The same tendency can be found of MLP. They both suggest that the sequence information is important for TAD boundaries’ formation.

The details of these results are available at https://github.com/LoopGan/TAD-Lactuca. To further test whether our model is cell-type and dataset specific, we applied TAD-Lactuca on other two datasets: hESC from Dixon [7] and IMR90 from Filippova [18]. The TAD-Lactuca also attained satisfactory results (Fig. 3b).

**Fig. 4:**
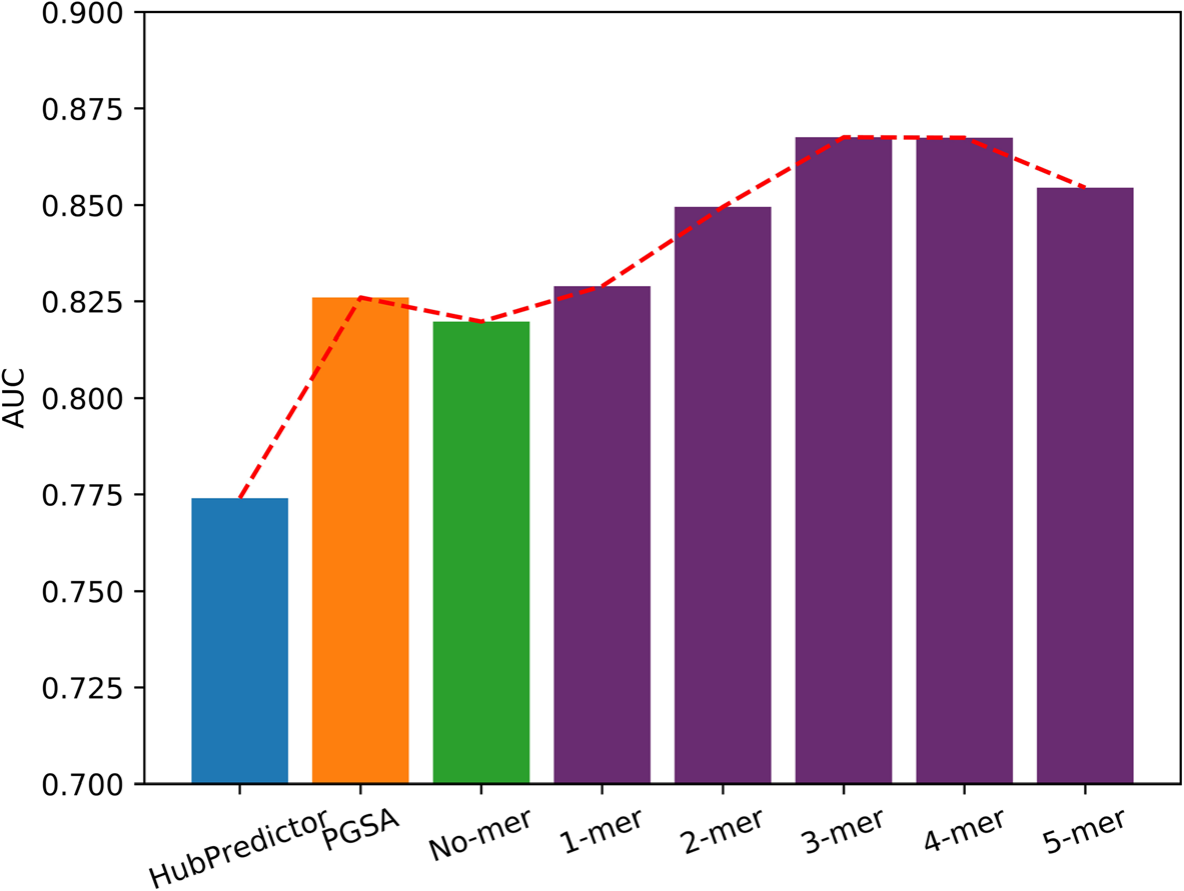
TAD boundary prediction compared with PGSA and HubPredictor. The HubPredictor bar (blue) shows the result by Huang [21], the PGSA bar (orange) shows the best multi-element models result by Hong [22]. The No-mer bar (green) shows the result of TAD-Lactuca without sequence information. The rest bar (purple) is the result of different k-mer combined with histone mark signals. The red dotted lines indicate their trend

When compared with PGSA [22], the performance of RF is a little worse while only taking the histone mark signals as features (Fig. 4). Significant improvement was observed when additional sequence information, particularly 3-mer features, combined. The performance improved with the length of k-mer increased. The length of the feature vector would increase sharply at the scale of 4^*k* _ *mer*^. Here, we only performed the experiment until k _ mer = 5, at which a performance decrease was observed.

Our two methods achieve a better performance than HubPredictor [21] and PGSA [22]. We attribute the results of models to the consideration of contextual and sequence information. Deep learning works excellent among mass of data. The data of our task is only about 4, 000, RF model with the highest performance is in our expectation.

### Robustness in different resolutions

**Fig. 5:**
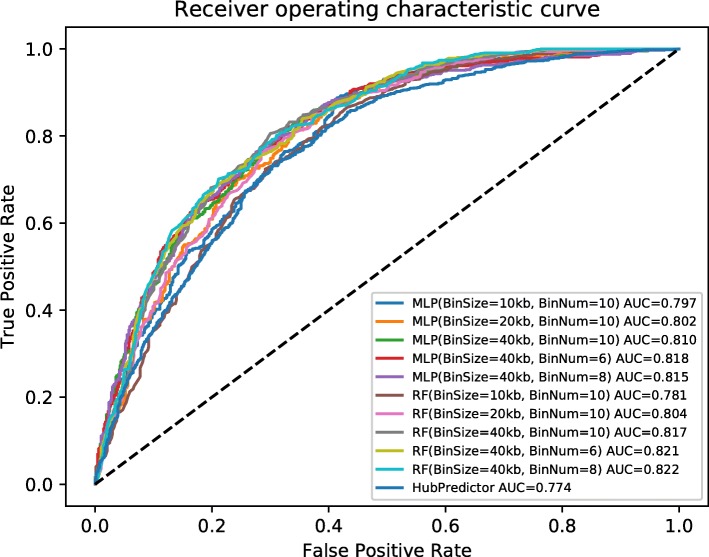
TAD–Lactuca has implemented Random Forests (RF) and Multi-Layer Perceptron (MLP) for different resolutions without sequence information. The ROC curves of different resolutions are shown, while the AUC scores and resolutions are shown in the rectangle

Resolution is a significant factor when identifying the TAD regions [24, 41]. We tested the robustness of TAD–Lactuca in different resolutions and adjusted the downstream and upstream bin number to 8 and 6. Furthermore, we also resized the bin to 20 kb and 10 kb. When we reduced the downstream or upstream region of the loci of interest, we found that TAD–Lactuca has an equal or even better performance in separating the TAD boundary from non-boundary. When we rescaled the size of the bin, the accuracy is approximately similar to that achieved with the bin sized 40 kb (Fig. 5). These results suggested that our method is robust at different resolutions. From Fig. 5, we also observed that TAD–Lactuca has better performance compared to HubPredictor [21] across all different resolutions.

## Discussion and conclusion

In this work, we designed the TAD–Lactuca to distinguish the TAD boundaries from other genomic areas by utilizing the CTCF and histone mark signals as well as sequence information around a locus of interest. It outperforms the existing methods in predicting the boundary of topologically-associated domains. We additionally applied our method on the hESC datasets produced by Dixon [7] and IMR90 dataset produced by Filippova [18] and then tested the TAD–Lactuca at various resolutions. All these results suggested the incorporation of sequence features could significantly improve the performance. The sequence motif enrichment analysis indicates several gene regulation motifs. It implies the sequence patterns would be important in chromatin folding.

Although TAD-Lactuca achieves good performance and detects several chromatin structure and gene regulatory associated motifs, there are some limitations in our approach. For example, the relationships between different histones not take into consideration, a model combined spatial information will be addressed in the future study.

## Materials and methods

### Materials

The TAD boundaries of IMR90 and hESC were obtained from Dixon [7], which is available from GEO with the accession number GSE35156. We also downloaded a contemporary dataset of IMR90 TAD boundaries from Filippova [18]. The TAD boundaries of these three datasets are provided as supplementary data. The genome-wide signal coverage tracks of CTCF for both cell types were downloaded from ENCODE [42], while the other eight histone mark (H3K4me1, H3K4me2, H3K4me3, H3K9ac, H3K9me3, H3K27ac, H3K27me3 and H3K36me3) signal tracks for the two cell types mentioned before were downloaded from NIH Roadmap Epigenome Project [43]. Due to the boundaries/non-boundaries’ coordinates basing on hg18, all these genome-wide signal coverage tracks were converted from hg19 to hg18 by the *lift* function of bwtool [44]. The k-mer frequency model is also based on hg18.

### Methods

**Fig. 6:**
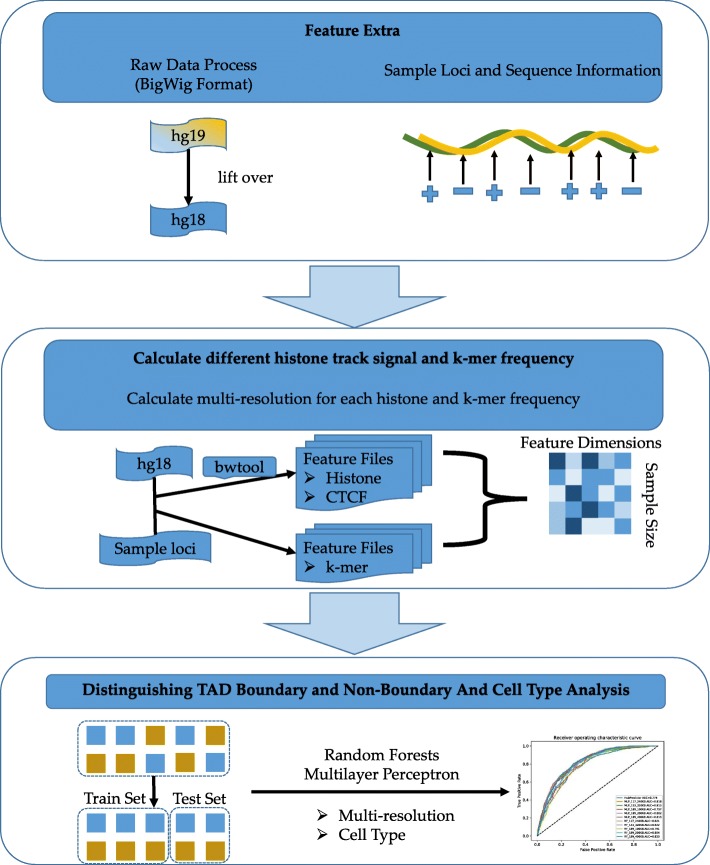
Three length k-mers. For *k* = 3, the first three k-mers are *GCA*, *CAA*, *AAC*, the rest and other length k-mer can be obtained as k-mer’s definition

Using the significant differences in CTCF and eight histone mark signals between TAD boundaries and the other regions, we proposed a method, TAD–Lactuca, for determining whether a locus on the genome is in a TAD boundary. To improve the prediction accuracy, the k-mer analysis merged into our model. The TAD–Lactuca used the signal intensity vector of CTCF and eight histone mark signals, different k-mer’s frequency for both the given locus and its context, respectively. These nine vectors were subsequently cascaded. While comparing with PGSA [44], the k-mer’s frequency vector also do the same operation. For positive samples, we directly used the TAD boundary downloaded from Dixon [7]. For negative samples, according to the method outlined previously [21], the same number of non-boundary genomic loci were randomly selected with a similar interaction frequency as the boundary. The TAD–Lactuca used the vector as input, before utilizing both the Random Forests model and Artificial Neural Network to fit the data. The workflow (Fig. 6) of TAD–Lactuca includes four steps: (1) downloading and processing data as previously mentioned; (2) selecting the loci as the description in the Pick Loci; (3) using bwtool [44] to get a 189-dimension (bin_size as [40 kb, 20 kb or 10 kb] respectively, bin_number as 10), a 153-dimension (bin_size as 40 kb, bin_number as 8) or a 117-dimension (bin_size as 40 kb, bin_number as 6) vector for each locus, calculating k-mer’s frequency for different k size(k as [1, 2, 3, 4 and 5]); and (4) letting TAD–Lactuca use a matrix of 4416 vectors of IMR90 (2208 positive samples and 2208 negative samples, with alternative other scales for hESC and contemporary IMR90 dataset [18]) as input to fit a model and provide predicted results.

### Pick loci

For the TAD boundaries of IMR90 and hESC, we selected the boundary loci from Dixon [7]. Dixon identified 2208 TAD boundaries of IMR90 and 3837 TAD boundaries of hESC by ‘DomainCaller’ [7], Filippova identified 4052 TAD boundaries by Armatus [18]. The non-boundary loci were randomly selected from the genomic loci with the same interaction frequency as the TAD boundaries [21]. For loci with several bins, the center bin would be taken as the region for the TAD boundaries’ or non-boundaries’ loci. The list of TAD boundary and non-boundary loci are available at https://github/LoopGan/TAD-Lactuca.

### Calculate signal

#### Histone signals

**Fig. 7:**
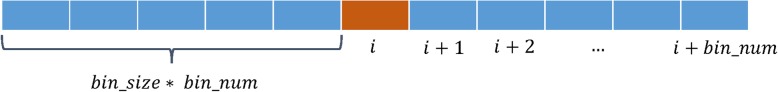
The workflow of the TAD–Lactuca. It contains two parts: Feature Extract and Model. The first part, namely feature extraction, can be divided into three parts: (1) obtaining raw data, (2) selecting loci and (3) calculating multi-resolution signals. The model part consists of two models named Random Forests (RF) and Multi-layer perceptron (MLP), which work individually and provide information regarding the locus of TAD boundaries

The patterns of different histone marks around TAD boundaries have been shown by previous studies [7, 21]. However, these previous studies [21] only used the averaged signal for each histone mark. In this present study, we argue that the context in which these signal patterns are found is also important for a boundary. So binning technology was introduced to describe the loci information. We take the up/downstream information to calculate CTCF and eight histone mark signals. As Fig. 7 shows, each bin contains *bin* _ *size* bases and *bin* _ *num* ∗ *bin* _ *size* ∗ 2 bases were considered. Then we used *summary* in bwtool [44] to calculate the signal intensity of the noticed factor in each bin. For example, *bin* _ *size* = 40kb (reported resolution of Hi–C experiment by Dixon [7]) and *bin* _ *num* = 10, we will get a vector lengthened to 21 to express a mark signals, not a scalar as previous done. For 9 different marks as we used, we will obtain a 21 * 9-dimension vector to describe a boundary/non-boundary. For CTCF and each histone mark signal, we alternatively calculated the mean signal for TAD boundaries and non-boundaries, with significant differences found in this study (Fig. 1).

#### DNA sequencing

**Fig. 8:**
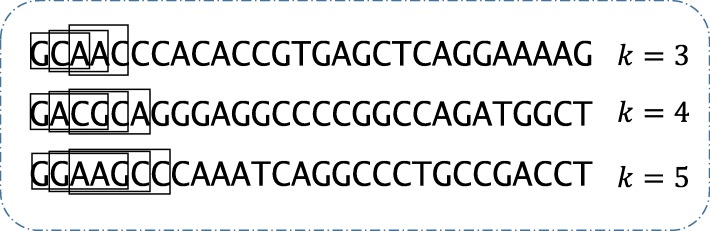
Bin *i* is the locus we want to know is a boundary or not. We take *bin* _ *size* ∗ *bin* _ *num* bases up/downstream to describe the locus

Letting *s* be a biological sequence of length *m*, then *s* = *q*_1_*q*_2_*q*_3_…*q*_*m*_, where *q*_*i*_ ∈ *Σ* (Σ is the symbol space of biological sequence, and for gene sequence, there is *Σ* = {*A*, *T*, *C*, *G*}). In bioinformatics, a consecutive symbol subsequence (of length k) starting at any position *i*(1 ≤ *i* ≤ *m* − *k* + 1) in a read is called k-mer. Different length k-mers can be obtained as Fig. 8 shown.

We use different k-mers’ frequencies as features to expression the sequence information of boundary/non-boundary. As so far, we only use the center bin’s sequence to calculate the different k-mer’s frequency. Take *k* = 3, *bin* _ *size* = 40*kb* as an example, we can get 64 useful k-mers, there may be some ‘N’ in the sequence, we just leave them alone and the sum of different k-mers’ frequency is less or equal than 1, so the feature vector is 64-dimension. Then we sort the k-mers by lexicographical, calculating the frequency as $Frequency(k)=\frac{\sum k- mer}{40000-3+1}$. For the model with sequence information, we concatenate the frequency vector at the tail of histone and CTCF marks signal vector.

### Model and measurement

The model of TAD–Lactuca contains two parts: Random Forests and Multi-Layer Perceptron. Random Forests and Multi-Layer Perceptron are all supervised learning techniques, which labels the target of each region as a boundary or non-boundary, 1 as boundaries and 0 as non-boundaries. The details of the two models are given in the following section.

Random Forests (RF) is a powerful machine-learning method developed by Breiman [45], which has been successfully and widely applied in epigenetics [7, 46, 47]. RF is a type of ensemble learning based on the decision tree. The algorithm to build the tree is the Classification and Regression Tree (CART). The RF contains two parts: (1) building the decision trees and (2) assembling the trees to form a forest for classification. The principles of RF are summarized briefly as follows:

- Build the decision trees

■ Sample N cases at random with replacements (i.e., a Bootstrap Sample method) to create a subset of the training set.

■ At each node:

◆ For some number m, m predictor variables are selected at random from all the M predictor variables. The value of m is constant during the forest growing.

◆ The predictor variables that provide the best split, according to some objective function, are used to do a binary split on the current node.

◆ At the next node, choose other m variables set at random from all predictor variables and repeat the first two steps until the node cannot be split or reach to a specific label.

◆ Each tree is grown to the largest extent possible, there is no pruning for the tree.

- Ensemble the trees to form a forest and provide classification

■ Repeat the *Build the decision trees* for some number of trees (T).

◆ Combine each tree (a weak learner) and form a forest (a strong learner).

◆ Each tree provides a classification and votes for a specific class. The forest chooses the classification with the most votes.

◆ Return the sample’s classification or label.

As a part of the TAD–Lactuca, we constructed a prediction model using the function *RandomForestClassifier* with default parameters, except for the *n_estimators* of sklearn [29], to quantitatively investigate the relationship between the signals of CTCF and histone mark with TAD boundaries. The value of the parameter *n_estimators* provides the number of trees in the forest. Considering the balance between the computing cost and the performance of the RF, we set *n* _ *estimators* = 500. The other parameters were searched by function *GridSearchCV* of sklearn [29]. For IMR90 from Dixon [7], the input of RF includes 2208 TAD boundaries and 2208 TAD non-boundaries. Cross-validation using the function *cross_val_score* of sklearn was used to estimate the performance of our RF model. To assess the performance of the features we have identified, we calculated the Area Under Curves (AUC), accuracy and F-score (See the *Supplementary Materials*), which are generated from 10-fold cross-validation.

Deep learning is another dominant technique in this classification task, which has been used in many fields, such as regulatory genomics and biological image analysis [48–50]. To the best of our knowledge, deep learning has not been used for TAD boundary prediction. In another part of TAD–Lactuca, we used the Multi-Layer Perceptron (MLP), a type of deep learning technique, in our method. A MLP might be viewed as a logistic regression classifier where the input is first transformed using a non-linear transformation. This transformation projects the input data into a space where it becomes linearly separable. Compared with a logistic regression classifier, the MLP fully utilizes the logistic regression classifier. It combines multiple logistic regression classifiers, before taking the former logistic regression classifier output as the input of the latter ones until the output layer of the MLP. The MLP overcomes the problem caused by the linear inseparability samples. It projects the samples into a feature space, before projecting the feature into the feature’s feature space until the last intermediate logistic regression is classified. These intermediate logistic regression classifiers are in a hidden layer. We provide a brief introduction about the MLP in the following section.

**Fig. 9:**
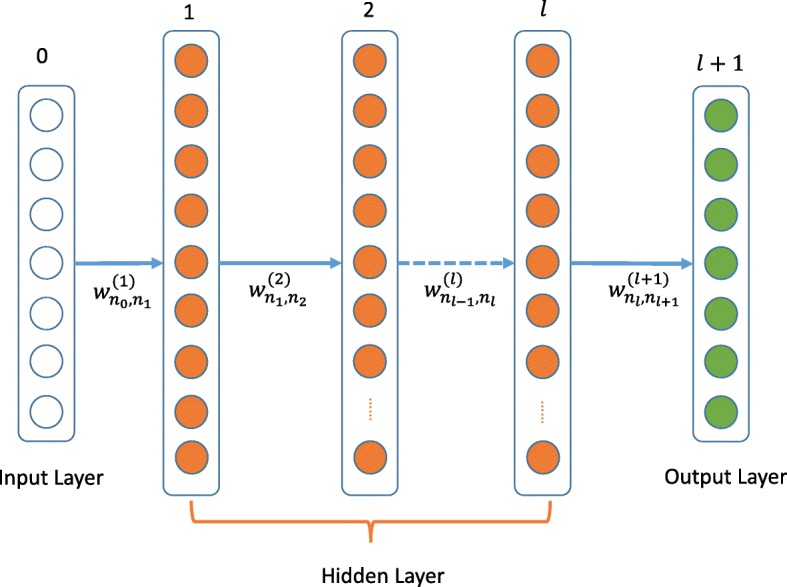
The diagrammatic sketch of MLP

**Fig. 10:**
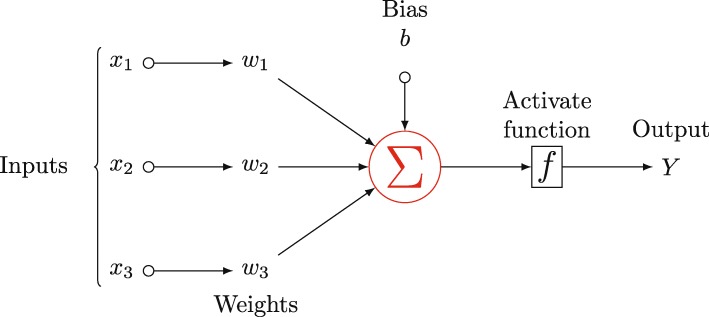
The output of a neuron

A MLP with *l* hidden layers is represented graphically in Fig. 9. The basic computing unit of an artificial neural network (ANN) is the neuron. The neuron in the input or output layer can be called as the input or output neurons. The MLP (demonstrated in Fig. 9) contains *n* input neurons, which creates n-dimensional features. The hidden layer contains vast neurons which were determined by model and the output layer often contains small quantity neurons. As our task is a binary classification problem, the output layer contains one neuron. For some neuron, the input and output are expressed as in Fig. 10.

The output Y can be calculated using Eq. (3):

3 $$ {\displaystyle \begin{array}{c} Output\kern0.17em of\kern0.17em neuron=Y\\ {}=f\left({\theta}_i^{\mathrm{T}}\overrightarrow{x}+{b}_i\right)\\ {}=f\left({w}_1{x}_1+{w}_2{x}_2+{w}_3{x}_3+b\right)\end{array}} $$

where the $\overrightarrow{x}$ represents the input of the neuron; the *θ*_*i*_ is the weight vector and the *b*_*i*_ is bias. For each layer, there is a special active function. In this study, the output neuron’s active function that we chose is *sigmoid*, which can be written as Eq. (4). The return value ranges from 0 to 1.

4 $$ S(x)=\frac{1}{1+{e}^{-x}} $$

where the *x* is the input of other neurons. The function will return a value. If the value is greater than a threshold, the input sample is labeled as a positive sample, otherwise it is labeled as a negative sample. The back propagation (BP) algorithm is used to train the neural network [51], in which the weight vector and bias can be updated by minimizing errors between the output and the true label.

**Fig. 11:**
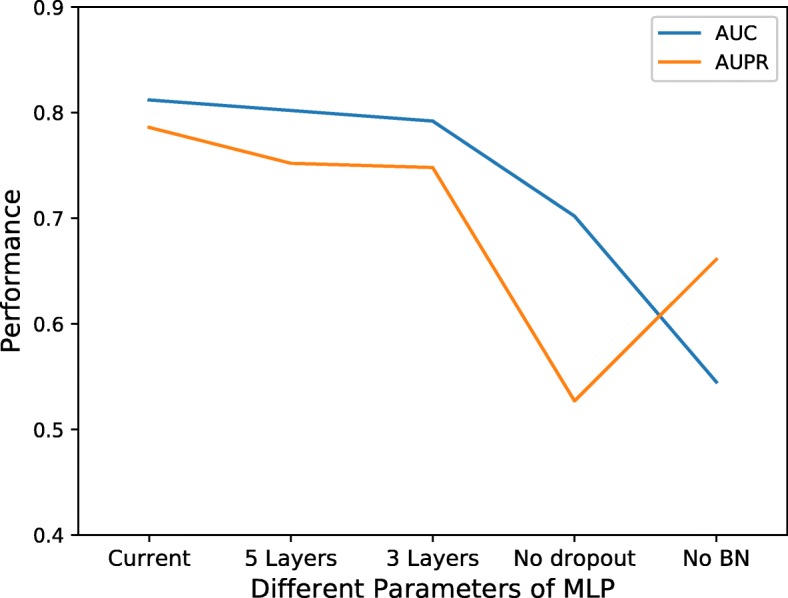
Different parameters of MLP performance. Current represents the parameters of MLP as Table 2 described. Compared with current, 5 Layers means there is double less Layer 2 and 3 Layers means Layer 2 is lacking. No dropout means each layer do not have dropout operation and No BN (Batch Normalization) means each layer are not normalized. They are all compared with Current situation

The MLP we used in this study contained four hidden layers. It is a 6-layer artificial neural network, implemented by Python with Tensorflow [52] and Keras [53]. For each layer, the number of neurons, active function and dropout size were searched by Hyperas and Hyperopt, Table 2 shows the details of the parameters and Fig. 11 shows AUC and AUPR values of MLP with other parameters.

**Table 2:** The parameters of MLP different layers. BN means Batch Normalization the input or not

	Number of Neurons	Active Function	DropOut	BN
Layer 1	512	linear	0.6975	No
Layer 2	256	softplus	0.5153	Yes
Layer 3	512	linear	0.4252	Yes
Layer 4	1024	hard_sigmoid	0	No

## Data Availability

A python 3.* implementation of the TAD–Lactuca and instructions for use are available at https://github.com/LoopGan/TAD-Lactuca. The origin data are available at https://github.com/LoopGan/TAD-Lactuca/tree/master/data.

## Abbreviations

3C-based: Chromatin Conformation Capture-Based
AUC: Area Under Curves
AURP: Area Under the Precision-Recall Curves
ChIP-seq: Chromatin Immunoprecipitation sequencing
CTCF: CCCTC-binding factor
Hi-C: High Throughput Chromosome Conformation Capture
ROC: Receiver Operating Characteristic Curve
TAD: Topologically Associating Domains
VDJ: Variable, diversity and joining genes
